# Human neural stem cells improve cognition and promote synaptic growth in two complementary transgenic models of Alzheimer's disease and neuronal loss

**DOI:** 10.1002/hipo.22405

**Published:** 2015-01-08

**Authors:** Rahasson R. Ager, Joy L. Davis, Andy Agazaryan, Francisca Benavente, Wayne W. Poon, Frank M. LaFerla, Mathew Blurton‐Jones

**Affiliations:** ^1^ Institute for Memory Impairments and Neurological DisordersUniversity of CaliforniaIrvineCalifornia; ^2^ Sue and Bill Gross Stem Cell Research CenterUniversity of CaliforniaIrvineCalifornia; ^3^ Department of Neurobiology and BehaviorUniversity of CaliforniaIrvineCalifornia

**Keywords:** Alzheimer's disease, neural stem cells, immune suppression, transplantation, therapeutic

## Abstract

Alzheimer's disease (AD) is the most prevalent age‐related neurodegenerative disorder, affecting over 35 million people worldwide. Pathologically, AD is characterized by the progressive accumulation of β‐amyloid (Aβ) plaques and neurofibrillary tangles within the brain. Together, these pathologies lead to marked neuronal and synaptic loss and corresponding impairments in cognition. Current treatments, and recent clinical trials, have failed to modify the clinical course of AD; thus, the development of novel and innovative therapies is urgently needed. Over the last decade, the potential use of stem cells to treat cognitive impairment has received growing attention. Specifically, neural stem cell transplantation as a treatment for AD offers a novel approach with tremendous therapeutic potential. We previously reported that intrahippocampal transplantation of murine neural stem cells (mNSCs) can enhance synaptogenesis and improve cognition in 3xTg‐AD mice and the CaM/Tet‐DT_A_ model of hippocampal neuronal loss. These promising findings prompted us to examine a human neural stem cell population, HuCNS‐SC, which has already been clinically tested for other neurodegenerative disorders. In this study, we provide the first evidence that transplantation of research grade HuCNS‐SCs can improve cognition in two complementary models of neurodegeneration. We also demonstrate that HuCNS‐SC cells can migrate and differentiate into immature neurons and glia and significantly increase synaptic and growth‐associated markers in both 3xTg‐AD and CaM/Tet‐DT_A_ mice. Interestingly, improvements in aged 3xTg‐AD mice were not associated with altered Aβ or tau pathology. Rather, our findings suggest that human NSC transplantation improves cognition by enhancing endogenous synaptogenesis. Taken together, our data provide the first preclinical evidence that human NSC transplantation could be a safe and effective therapeutic approach for treating AD. © 2014 The Authors. Hippocampus Published by Wiley Periodicals, Inc.

## INTRODUCTION

Alzheimer's disease is the leading cause of dementia, affecting over 35 million people worldwide, and the prevalence of AD is expected to more than double by 2030 (Wimo and Prince, 2010). AD is first diagnosed by the appearance of several well‐characterized symptoms, including short‐term memory loss, learning impairments, and disruptions in daily activities (Burns and Iliffe, 2009). Pathologically, AD is defined by two hallmark lesions, Aβ plaques and tau‐laden neurofibrillary tangles that are accompanied by inflammation and extensive neuronal and synaptic loss (Querfurth and LaFerla, 2010). The degree of cognitive disruption associated with AD is largely attributed to the widespread neuronal and synaptic loss that affects multiple networks within the brain (Terry et al., 1991). Unfortunately, current therapies provide only modest symptomatic relief and fail to modify the long‐term progression of this disease.

Over the past decade, significant effort has been put forth to identify and develop therapies that reverse or prevent the accumulation of AD‐associated pathology. The primary molecular target of such research has been the Aβ peptide; based on considerable evidence that Aβ accumulation drives many of the downstream pathologies associated with AD (Hardy and Selkoe, 2002; Tanzi and Bertram, 2005; Citron, 2010). Preclinical mouse studies have suggested that single targeted approaches against Aβ, particularly using immunotherapy, might be effective. However, thus far, such therapies have provided little to no clinical improvements in human trials, despite evidence of reduced Aβ load (Karran et al., 2011). The lack of success of Aβ–targeting therapies suggest that a single‐pronged approach to the treatment of AD may be insufficient and has prompted an increased focus on the development of alternative methods that engage multiple mechanisms of action and target other downstream pathways such as synaptogenesis (Schubert and Maher, 2012).

For over 20 yrs, it has been known that synaptic loss in AD correlates more closely with cognitive impairment than either Aβ or tau pathology (Terry et al., 1991; Selkoe, 2002). Hence, treatments that promote the regeneration, replacement, or repair of lost or damaged synapses might provide a meaningful clinical benefit. Over the past decade, stem cell‐based therapies have grown to represent a promising treatment strategy for many central nervous system (CNS) disorders (Kiskinis and Eggan, 2010; Lindvall and Kokaia, 2010). Such therapies could offer the advantage of targeting multiple mechanisms, which, alone or in combination, could include the replacement of lost cells, neuroprotection through trophic factor secretion, or modulation of inflammation. Multiple studies using murine neural stem cells have provided proof of concept demonstrating benefits to motor and cognitive function after CNS injury (Riess et al., 2002; Jeltsch et al., 2003; Shear et al., 2004). Human stem cell transplantation has also been shown to be beneficial in rodent models of CNS trauma and chronic neurodegeneration (Kelly et al., 2004; Cummings et al., 2005; Keirstead et al., 2005; Tate et al., 2010; Rosenkranz et al., 2012). The types of stem cells used thus far have originated from a variety of sources, including the CNS, bone marrow, and adipose tissues. Regardless of the donor cell type, these studies and others have found that a leading beneficial effect of stem cell transplantation is due to the secretion of trophic factors, a mechanism that is often referred to as a bystander or neuroprotective effect (Martino and Pluchino, 2006; Shimada and Spees, 2011).

Our lab previously reported that murine NSC (mNSC) transplantation improved cognition in mouse models of neurodegeneration applicable to AD (Yamasaki et al., 2007; Blurton‐Jones et al., 2009). To successfully transition a stem‐cell based approach into a clinical application; a suitable human stem cell line must first be identified and tested in preclinical studies, using the most relevant models of AD. To that end, in the current study, we tested whether the cognitive dysfunction resulting from the loss of hippocampal CA1 neurons (CaM/Tet‐DT_A_) or Aβ and tau accumulation (3xTg‐AD), could be rescued by human CNS stem cells (HuCNS‐SC) transplantation. In addition, we sought to determine whether HuCNS‐SC transplantation could modulate either Aβ and tau pathology and/or markers of synaptic growth and plasticity.

To achieve robust xenograft survival in these two animal models, different immunosuppression regimens were used. For CaM/Tet‐DT_A_ mice, immunosuppression was achieved with the calcineurin inhibitor FK506, in combination with an antibody against CD4. Given the impact of FK506 on tau pathology (Yoshiyama et al., 2007), an alternative immunosuppression regimen was used for 3xTg‐AD mice that target the T‐cell costimulatory receptors LFA‐1, CD40L, and CTLA‐4 (Pearl et al., 2011). In both models, we found that human NSCs survive, migrate, and differentiate within the host hippocampus leading to significant improvements in cognitive function. Examination of markers of structural and synaptic plasticity also provides intriguing evidence that NSCs likely improve cognition by enhancing endogenous neuronal connectivity. To our knowledge, this is the first report demonstrating therapeutic efficacy of clinically relevant human CNS stem cells in two separate and complimentary models of AD neurodegeneration.

## MATERIALS AND METHODS

### Animal Models

The 3xTg‐AD mice harbor the human APP‐Swedish mutation (KM670/671NL), tau mutation (P301L), and presenilin‐1 mutation (M146V) and are maintained on a C57BL6/129 background (Oddo et al., 2003). The double transgenic CaM/Tet‐DT_A_ mice, maintained on a (B6/CBA) background, represent a model of hippocampal neurodegeneration with significant loss of CA1 pyramidal neurons; have also previously been characterized (Yamasaki et al., 2007). To induce neuronal loss, doxycycline‐enriched chow (Research Diets, NJ) was removed from the diet of CaM/Tet‐DT_A_ mice and replaced with normal chow, leading to the expression of diphtheria A chain within hippocampal CA1 neurons. Placing mice back onto a doxycycline diet after 25 days of DT_A_ induction halted further neuronal loss. Both male and female double transgenic CaM/Tet‐DT_A_ mice and single transgenic Tet‐DT_A_ mice, treated at 7 months of age, and female 3xTg‐AD mice, treated at 19 months, were used for this study. All animal procedures were performed in strict accordance to the guidelines of the University of California, Irvine, Institutional Animal Care and Use Committee.

### Isolation and Expansion of Human‐CNS Derived Stem Cells

Research grade HuCNS‐SC cells were isolated as previously described (Uchida et al., 2000) and generously provided by Stem Cells Inc. In brief, CD133^+^ CD24^−/low^ cells were allowed to form neurospheres after 10–14 days in culture. Cells were passaged every 10–14 days into single cell suspensions before being cryopreserved. Importantly, previous reports have shown that HuCNS‐SCs produce physiologically relevant levels of brain‐derived neurotrophic factor (BDNF) (McGill et al., 2012), a factor previously implicated in our studies of murine NSC transplantation (Blurton‐Jones et al., 2009). Before *in vivo* experiments, frozen cells were thawed and further expanded as neurospheres. Before transplantation, cells were harvested, counted, and viability determined. A total of 100,000 live cells per hippocampus were transplanted over the course of 4 min at a concentration of 5 × 10^4^ cells per microliter. Viability of cells at the time of transplantation was >93% (data not shown).

### Transplantation Surgery

For transplantation surgeries, mice were anesthetized with isoflurane (Western Medical Supply, CA), and placed into a Kopf stereotaxic frame. Normal body temperature was maintained using an automated thermoregulation system (KOPF Instruments, CA). HuCNS‐SC, at 1.0 × 10^5^ cells per site (2 μL/injection), or vehicle, were injected bilaterally into the hippocampus at a rate of 1 µL/min using the following coordinates relative to bregma: anteroposterior (A/P): −2.06 mm; dorsoventral (D/V): −1.95 mm; mediolateral (M/L): ±1.75 mm. Transplantation of HuCNS‐SC into 3xTg‐AD mice used identical procedures and coordinates. After surgery, the incision was sealed with Tissuemend II (Western Medical Supply, CA) and topical antibiotic ointment applied before allowing mice to recover on heated pads.

### Immunosuppression

The commonly used immunosuppressants cyclosporine and FK‐506 can modulate AD pathology (Yu et al., 2006; Yoshiyama et al., 2007; Hong et al., 2010); consequently, we used a recently developed immune suppression paradigm to target leukocyte costimulatory molecules and allow xenogeneic stem cell engraftment in 3xTg‐AD mice (Pearl et al., 2011). Both vehicle‐injected, and HuCNS‐SC‐transplanted 3xTg‐AD mice were immunosuppressed by intraperitoneal (i.p.) injection of anti‐LFA‐1 (20 mg/kg), anti‐CD40L (20 mg/kg), and h‐CTLA‐4‐Ig fusion protein (20 mg/kg) (BioXcell, West Lebanon, NH) on the day of transplantation and days 2,4, and 6 post‐transplantation.

CaM/Tet‐DT_A_ mice were immunosuppressed with a combination of FK506 (5 mg/kg; Sigma Aldrich, MO), i.p. daily, beginning 3 days before transplantation, and anti‐CD4 (20 mg/kg; BioXcell), i.p. beginning the day of transplantation and lasting for 4 consecutive days and repeated every 7 days thereafter.

### Behavioral Testing

The 3xTg‐AD mice were trained using the Morris water maze (MWM) and novel object recognition (NOR) task to assess hippocampal‐dependent learning and memory beginning 4 weeks post‐HuCNS‐SC transplantation. All mice were initially hand habituated 3 days before behavioral assessment. Both tasks were conducted as previously described (Blurton‐Jones et al., 2009). For MWM, the task was run in a 1‐m diameter circular pool filled with opaque water at 25°C. Mice were trained to swim to a 14‐cm diameter circular platform submerged 1.5 cm beneath the surface of the water and invisible to the mice. Mice were subjected to four trials per day. During each trial, mice were placed into the tank at one of four designated start points in a pseudorandom order. Mice were trained until they reached a training criterion of 25 s (escape latency). To determine spatial memory retention, mice were tested in a probe trial 24 h after the final training session. Performance was monitored with the EthoVision XT video tracking system (Noldus, WA).

For both 3xTg‐AD and CaM/Tet‐DT_A_ mice, context‐dependent and place‐dependent versions of the novel object recognition (NOR) task were performed following standard protocols (Mumby et al., 2002; Yamasaki et al., 2007). During training, mice were exposed to two identical objects for 5 min in a round container with bedding and then subsequently a different pair of identical objects for another 5 min in a rectangular container with bedding. To assess memory retention, mice were placed 24 h later into either the round or rectangular cage where a familiar object was replaced with a novel object for that context. The percentage of time spent exploring the out‐of‐context novel object vs. the total time exploring both objects was calculated as the context‐dependent recognition index. Place‐dependent NOR also followed established protocols (Mumby et al., 2002; Yamasaki et al., 2007). In brief, mice were exposed to two identical objects for 5 min in a rectangular container with bedding. 24 h later, mice were placed back into the rectangular container where one object had been moved to a new location. The percentage of time spent exploring the out‐of‐place object vs. the total time exploring both objects was calculated as the place‐dependent recognition index.

### Tissue Preparation

After the 2 weeks of behavioral testing, mice were anesthetized with euthasol (0.5 μL/μg, Virbac Animal Health) then perfused transcardially with cold phosphate‐buffered saline (PBS). Brains were rapidly removed, cut along the midsagittal plane, and half the brain was immediately frozen and the other half drop‐fixed in 4% paraformaldehyde for 48 h. Fixed tissue was then cryoprotected in 30% sucrose, cut in 40 µm coronal sections on a freezing microtome, and stored at 4°C in PBS with 0.02% sodium azide. Frozen tissue was homogenized using a sonic dismembrator (Fisher Scientific) in T‐PER reagent (Thermo Scientific) with protease inhibitor cocktail (Roche Diagnostics). Homogenates were then centrifuged for 1 h at 100,000*g*, 4°C. The resulting supernatant was collected as the detergent‐soluble fraction. The pellet was then resuspended in 70% formic acid, centrifuged at 100,000*g* for 1 h, and the supernatant collected as the detergent‐insoluble fraction.

### Stereological Assessment

Unbiased stereological analysis of the hippocampal formation was conducted as previously described (Baglietto‐Vargas et al., 2010). Sections were examined on an Olympus BX61 microscope and analyzed using the optical fractionator probe and a Stereo Investigator system with MicroLucida (MBF Biosciences). The number of engrafted HuCNS‐SC was quantified in every seventh coronal section with a distance of 280 µm between sections using a 100X/1.35 objective throughout the entire anterior/posterior extent of the hippocampus (Franklin and Paxinos, 1997). The counting frame area was set at 3,600 µm^2^, with step lengths of 120 × 120 µm. Total cell number was estimated using the optical fractionator formula, *N* = 1/ssf.1/asf.1/hsf.∑Q−, where ssf represents the section sampling fraction, asf is the area sampling fraction, hsf is the height sampling fraction, and ∑Q− is the total count of nuclei sampled for the entire layer (West, 1999; Dorph‐Petersen et al., 2001; Schmitz and Hof, 2005). The precision of the individual estimations was expressed by the total coefficient of error (CE) (Gundersen et al., 1999).

### Immunostaining and Image Analysis

Immunohistochemistry was performed on free‐floating 40‐μm‐thick coronal sections. For light‐level Diaminobenzidine (DAB) staining, endogenous peroxidase was blocked with 3% H2O2/10% methanol in PBS and nonspecific binding blocked by incubation in blocking solution [5% normal horse sera, 3% bovine serum albumin (BSA) +0.1% Triton in PBS]. Sections were then incubated with primary antibody (overnight, 4°C) in blocking solution followed by the corresponding biotinylated secondary antibodies (1 h, 25°C) and then avidin–biotin–peroxidase solution (Vector Labs). Staining was visualized with DAB. Sections were then mounted on slides, dehydrated, and coverslipped.

For fluorescent immunostaining, free‐floating sections were blocked for 1 h (5% Donkey serum, 0.2% Triton, in PBS) and then incubated in primary antibody diluted in blocking solution (overnight, 4°C). After rinses, sections were incubated with appropriate Alexa conjugated‐secondary antibodies (Invitrogen) for 1 h and then rinsed. Sections were then mounted and coverslipped with Fluoromount‐G (Southern Biotech). The following primary antibodies were used: anti‐PHF1 (Thermo Scientific), anti‐human nuclei antigen (EMD Millipore), anti‐GFAP (Dako), anti‐oligodendrocyte specific protein (Abcam), anti‐doublecortin (Santa Cruz Biotechnology), STEM121^TM^‐anti‐human cytoplasm antibody (Stem Cell Sciences), Ku80‐anti‐human nuclei antibody (Cell Signaling), anti‐synapsin (EMB Millipore), anti‐synaptophysin (Sigma Aldrich), anti‐GAP43 (Sigma Aldrich), and anti‐Poly‐Tau (Dako). Fluorescent sections were imaged with a Leica DM 2500 laser scanning confocal or Zeiss 510 Meta confocal. All images represent either single confocal Z‐slices or Z‐stacks. For quantitative analysis, slides were coded and then images captured by a blinded observer using a Zeiss 510 Meta confocal microscope with identical laser and detection settings. Grayscale Z‐stack images were analyzed using Image J software. Four square regions of interest (ROIs) were randomly defined within the stratum radiatum of CA1 and the mean pixel intensity computed for each ROI. The four fields were averaged for each sample and means were statistically compared with analysis of variance and *post hoc* tests (ANOVA, Fisher's PLSD).

### ELISA Assays for Soluble and Insoluble Aβ_40_ and Aβ_42_

Aβ levels were quantified using a sensitive sandwich ELISA as previously described (Blurton‐Jones et al., 2009). In brief, immulon 2HB flat‐bottom wells (Thermo Scientific) were coated overnight at 4°C with monoclonal anti‐Aβ_1–16_ (generous gift of Dr. W. van Nostrand, Stony Brook University) at 30 μg/mL in 0.1*M* carbonate buffer (pH 9.6). Wells were then blocked with 3% BSA in PBS at 37°C for 3 h. Next, plates were washed and wells loaded in triplicate with brain homogenates or human Aβ_40_ or Aβ_42_ standards (EMD Millipore) diluted in capture buffer (20 m*M* sodium phosphate, 0.4*M* NaCl, 2 m*M* EDTA, 0.3% BSA, 0.05% CHAPS, and 0.05% Na azide, pH 7.0) and incubated overnight at 4°C. Plates were washed again and then probed overnight at 4°C with either biotinylated monoclonal anti‐Aβ_40_ or anti‐Aβ_42_. After washes, wells were incubated with streptavidin‐HRP (Thermo Scientific) for 3 h at 37°C. Plates were then developed with Ultra TMB‐ELISA substrate (Thermo Scientific) followed by 0.8*M* Ο‐phosphoric acid to stop the reaction. Plates were then read at 450 nm using a microplate photometer (Labsystems).

### Statistical Analysis

Multiple comparisons were performed using analysis of variance (ANOVA) followed by Fisher's Partial Least‐Squares Difference (PLSD) *post hoc* analysis. A repeated‐measures ANOVA was used to analyze MWM learning curve. Comparisons of two groups used students paired *t*‐test. Significance was set at 95% of confidence (*P* < 0.05) and all values are presented as mean ± SEM.

## RESULTS

### Human NSC Transplantation Ameliorates Context and Spatial Learning and Memory Impairments in Transgenic Models of AD and Neurodegeneration

Aged 3xTg‐AD mice exhibit extensive Aβ and tau pathology, and cognitive dysfunction, mimicking the hallmark pathologies of AD (Supporting Information Fig. S1A). However, most transgenic AD models lack substantial neuronal loss, a shortcoming that may explain recent failures in the translation of preclinical results (Zahs and Ashe, 2010). To better mimic the marked loss of hippocampal neurons that occurs in AD, we previously developed a transgenic model of hippocampal neuronal loss based on the tetracycline‐off inducible system (Yamasaki et al., 2007). Neuronal loss in this model is restricted to CaMKIIα‐expressing cells and is initiated via the withdrawal of doxycycline from the diet, leading to expression of the diphtheria toxin A chain (DT_A_) within these neurons. Withdrawal of doxycycline for a period of 25 days results in significant reductions in the neuronal marker NeuN in hippocampal CA1 subfield, and behavioral impairments, in double transgenic lesion vs. noninduced single transgenic controls (Supporting Information Fig. S1B).

**Figure 1:**
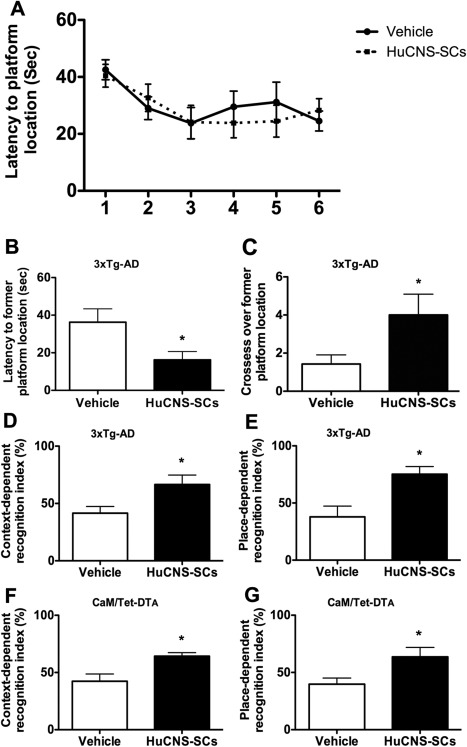
Transplantation of HuCNS‐SC improves learning and memory in two complementary models of AD pathogenesis and hippocampal neuronal loss. One‐month after transplantation 3xTg‐AD mice were tested on three independent cognitive tasks. Although no differences were detected in Morris water maze (MWM) acquisition (A), HuCNS‐SC transplantation (black boxes) significantly improved spatial memory as evidenced by decreased probe trail latencies (B) and increased platform crosses (C) vs. vehicle‐treated (white boxes) transgenics (ANOVA *P* < 0.05, Fisher's PLSD *P* = 0.034 and *P* = 0.05). (D) Performance in context‐recognition and (E) place recognition was also significantly improved by HuCNS‐SC transplantation (ANOVA *P* < 0.05, Fisher's PLSD *P* = 0.029 and *P* = 0.01). Beginning 1‐month after transplantation, CaM/Tet‐DT_A_ mice were also tested in context‐ (F) and place‐recognition (G) tasks. HuCNS‐SC transplantation significantly improved performance of lesioned mice vs. vehicle‐injected lesioned mice in both tasks (context: recognition index = 64.3 vs. 42.26, ANOVA *P* < 0.05, Fisher's PLSD *P* = 0.04; place: recognition index = 58.87 vs. 39.8, ANOVA *P* < 0.05, Fisher's PLSD *P* = 0.04). Data presented as mean ± SEM.

To determine whether human NSC transplantation could rescue the cognitive impairment associated with hippocampal neuronal loss or age‐dependent Aβ and tau accumulation, immunosuppressed CaM/Tet‐DT_A_ and 3xTg‐AD mice were treated with HuCNS‐SC or vehicle and tested in a series of context‐ and spatial‐dependent behavioral tasks beginning 4 weeks after transplantation. To assess changes in spatial learning and memory, 3xTg‐AD mice were first tested on the hidden platform MWM task. Mice were trained using four trials per day until a learning plateau was reached for both vehicle and HuCNS‐SC transplanted groups. No significant difference in MWM acquisition was observed between 3xTg‐AD‐vehicle and 3xTg‐AD‐HuCNS‐SC groups (Fig. 1A). However, 3xTg‐AD‐HuCNS‐SC mice performed significantly better during the probe trial, administered 24 h after the final training trial. During the probe trial, 3xTg‐AD‐HuCNS‐SC mice reached the former location of the hidden platform more than twice as fast as vehicle‐injected transgenic mice (16.3 ± 4.04 s vs. 36.3 ± 7.0 s, ANOVA *P* < 0.05, Fisher's PLSD *P* = 0.034) (Fig. 1B). The 3xTg‐AD mice treated with HuCNS‐SC also crossed the former platform location almost three times as often as vehicle‐injected transgenic mice, suggesting that HuCNS‐SC‐treated mice had formed a stronger memory for the platform's location (4.0 ± 1.0 vs. 1.4 ± 0.4 times, ANOVA *P* < 0.05, Fisher's PLSD *P* = 0.05) (Fig. 1C).

In addition to MWM assessment, both 3xTg‐AD and CaM/Tet‐DT_A_ mice were tested in context and place‐dependent versions of a novel object recognition (NOR) task. In the context‐dependent NOR task, mice are required to remember which objects are associated with a specific context (i.e., round vs. square arena). In the place‐dependent task, mice are expected to recognize which of two identical objects has been moved between training and test phases. When the performance of 3xTg‐AD mice were compared in context‐dependent NOR, 3xTg‐AD‐HuCNS‐SC injected mice spent significantly more time with the object placed into a new context vs. 3xTg‐AD‐vehicle mice (recognition index= 66.54 ± 8.2 s vs. 41.55 ± 5.7 s, ANOVA *P* < 0.05, Fisher's PLSD *P* = 0.029) (Fig. 1D). A similar difference in the capacity of 3xTg‐AD mice to recognize the novel placement of an object was also detected in the place‐dependent NOR task (recognition index HuCNS‐SC = 72.56 ± 6.7 s vs. Vehicle = 37.85 ± 9.3 s, ANOVA *P* < 0.05, Fisher's PLSD *P* = 0.01) (Fig. 1E). Importantly, no group differences in total exploration time were observed, suggesting there is no change in either motor function or general exploratory behavior (data not shown). Thus, HuCNS‐SC transplantation improves 3xTg‐AD cognitive function in three independent memory tasks.

CaM/Tet‐DT_A_ mice were also examined using context‐ and place‐dependent NOR. As shown, lesioned CaM/Tet‐DT_A_ mice that received bilateral HuCNS‐SC transplants spent significantly more time with the out of context object than vehicle‐injected lesioned mice (recognition index = 64.3 ± 3.1 s vs. 42.26 ± 6.3 s, ANOVA *P* < 0.05, Fisher's PLSD *P* = 0.04) (Fig. 1F). Similarly, HuCNS‐SC transplantation also significantly enhanced place‐dependent memory performance (recognition index = 63.6 ± 8.2 s vs. 39.8 ± 5.2 s, ANOVA *P* < 0.05, Fisher's PLSD *P* = 0.04) (Fig. 1G). Importantly, no group differences in total exploration time were observed for either context‐ and place‐dependent NOR (data not shown). Thus, HuCNS‐SC transplantation can improve memory in a model that mimics the extensive loss of hippocampal neurons that occurs in AD.

### Survival, Differentiation, and Migration of HuCNS‐SC Cells

**Figure 2:**
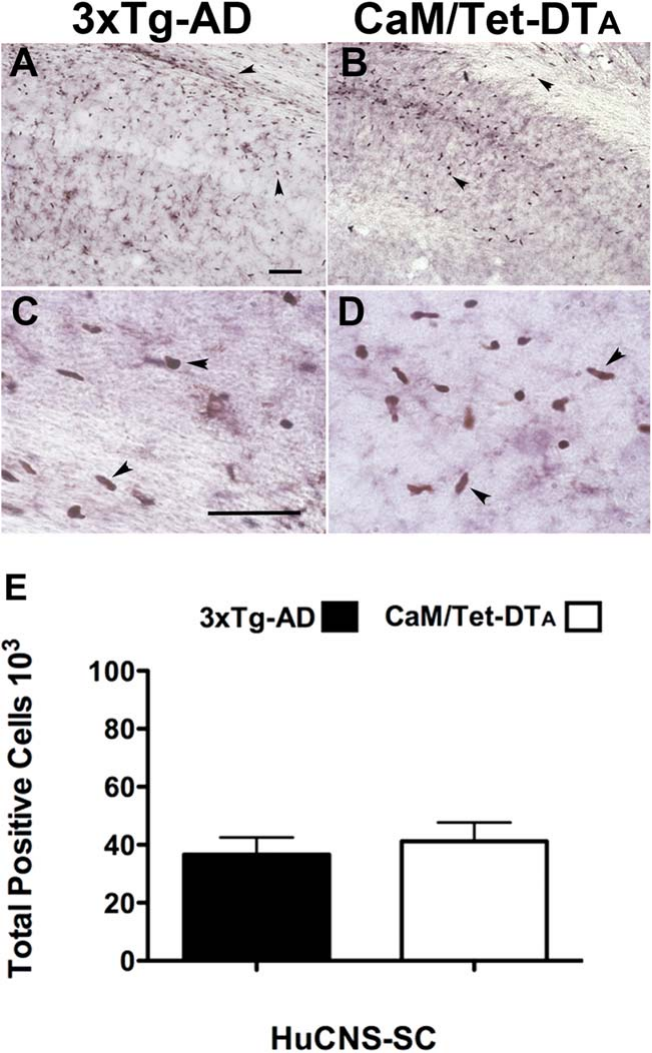
Both novel and traditional immune suppression paradigms allow robust survival and engraftment of human Neural Stem Cells. Given the reported effects of calcineurin inhibitors on AD pathogenesis, we used a novel paradigm that targets leukocyte costimulatory molecules to achieve xenotransplantation in 3xTg‐AD mice. (A) 6 weeks after transplantation, engrafted HuCNS‐SC (human nuclear antigen), were located within the hippocampus and overlying corpus collosum. (B) Immunosuppression with FK506 and anti‐CD4 provided a similar degree of HuCNS‐SC engraftment in CaM/Tet‐DT_A_ mice. (C, D) High power images from subfields of A and B reveal HuCNS‐SC positive for human nuclear antigen (brown). (E) Unbiased stereological assessment of HuCNS‐SC engraftment demonstrates that approximately 40,000 transplanted cells survive per injection site in both 3xTg‐AD and CaM/Tet‐DT_A_ models (optical fractionator probe, *N* = 6). Data presented as mean ± SEM. Scale bars = 50 μm. [Color figure can be viewed in the online issue, which is available at wileyonlinelibrary.com.]

To investigate the survival, differentiation, and migration of transplanted HuCNS‐SC, all mice were sacrificed after the 2 weeks of behavioral testing, such that the brains were examined 6 weeks post‐transplantation. Given growing evidence that commonly used immunosuppressant drugs such as the calcineurin inhibitors, can modulate AD‐associated pathologies, 3xTg‐AD mice were immunosuppressed with a recently described recombinant antibody approach that targets the T‐cell costimulatory molecules, LFA‐1, CD40, and CTLA‐4 (Pearl et al., 2011). We found that this immunosuppression paradigm led to robust engraftment and survival of human cells (Figs. 2A,C) even in mouse models with significant neuropathology. Similarly, immunosuppression of CaM/Tet‐DT_A_ mice with anti‐CD4 antibody and FK506 provided a similar degree of HuCNS‐SC engraftment (Figs. 2B,D). As expected, no HuCNS‐SC were detected in vehicle‐treated controls of either model (data not shown). Engrafted HuCNS‐SC were primarily found within and surrounding the CA1 subfield of the hippocampus, and also within the overlying corpus callosum (Fig. 2).

To quantify the number of surviving HuCNS‐SC, coronal slices representing the total anterior–posterior axis of the hippocampus were stained with an antibody against a human nuclear antigen and quantified by stereology using the optical fractionator probe. A total of 6 weeks post‐transplantation, an average of 36,600 cells per hippocampi had survived within 3xTg‐AD mice, and 41,170 cells survived within CaM/Tet‐DT_A_ mice (Fig. 2E). Stereological estimation of HuCNS‐SC engraftment therefore revealed substantial survival of transplanted human cells in two distinct immunosuppressed transgenic models of AD‐associated neurodegeneration.

To determine the fate of surviving HuCNS‐SC, we investigated the differentiation of HuCNS‐SC using immunofluorescence and confocal microscopy. The differentiation profile of transplanted HuCNS‐SC seemed to be largely dependent on the location and density of engrafted cells. First, we investigated whether some portion of the surviving HuCNS‐SC maintained expression of neural stem cell markers. Using an antibody against nestin, we found that a large proportion of HuCNS‐SC were positive for nestin in both transgenic models (data not shown). These findings are in accordance with previous studies demonstrating that human NSCs often take considerable time to differentiate after transplantation (Naimi et al., 1996).

**Figure 3:**
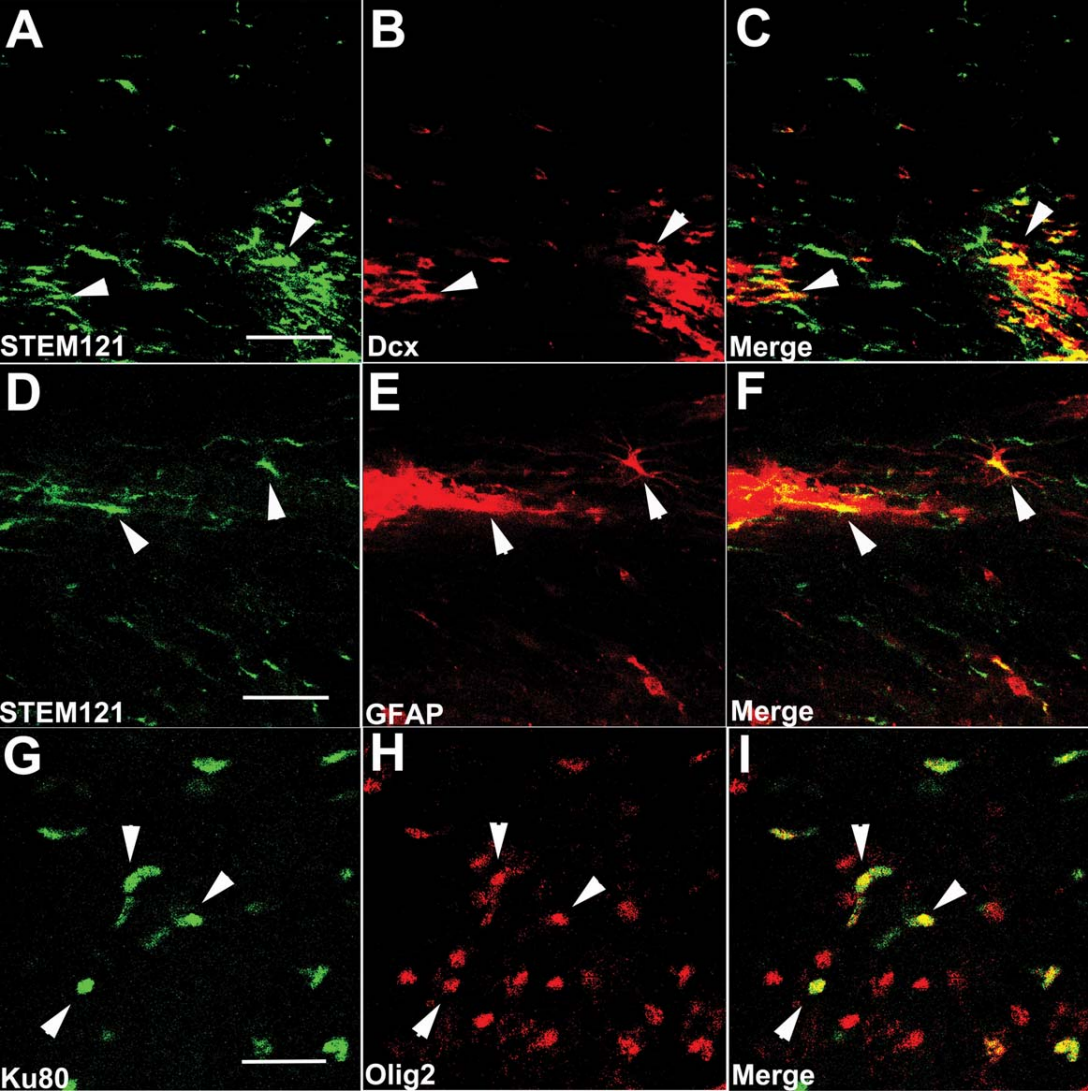
HuCNS‐SC show initial signs of differentiation in 3xTg‐AD mice. (A–C) HuCNS‐SC (green) injected into the hippocampus express doublecortin (red), a marker of immature neurons, 6 weeks after transplantation. (D–F) Examples of engrafted HuCNS‐SC (green) that coexpress the astrocytic marker GFAP (red) were also observed. (G–I) HuCNS‐SC expressing the nuclear marker Ku80 (green) also coexpress the immature oligodendrocyte marker olig‐2 (red). Scale bars, 25 μm. [Color figure can be viewed in the online issue, which is available at wileyonlinelibrary.com.]

**Figure 4:**
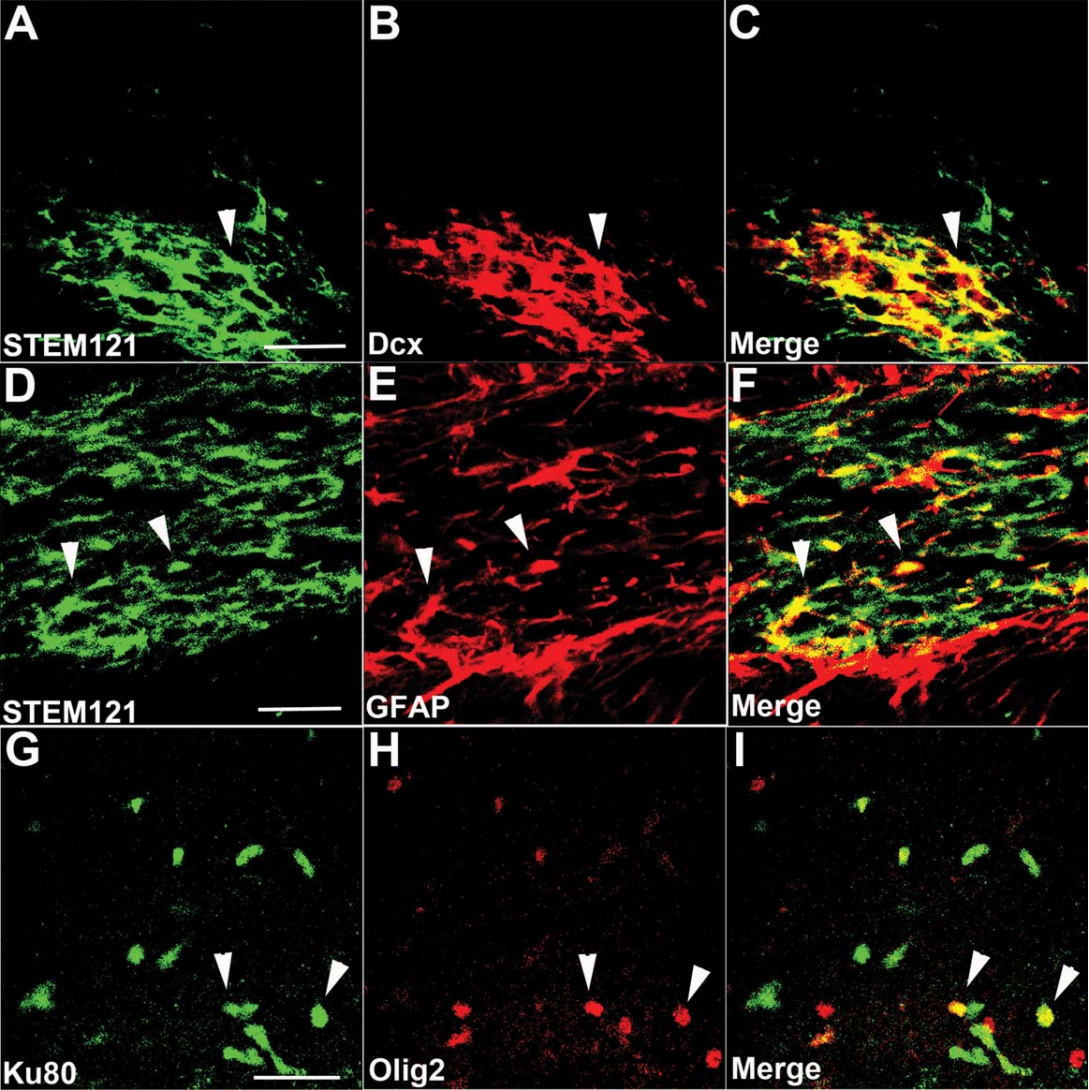
HuCNS‐SCs transplanted into CaM/Tet‐DT_A_ mice also exhibit evidence of neural and glial differentiation. (A–C) HuCNS‐SCs (green) injected into lesioned CaM/Tet‐DT_A_ mice often co‐expressed the early neuronal marker doublecortin (red). (D–F) Examples of HuCNS‐SCs (green) that colocalized with GFAP (red) were also detected. (G–I) In accordance with our findings in 3xTg‐AD mice, HuCNS‐SCs (green) were also found to coexpress the oligodendroglial marker olig‐2 (red). Scale bars, 25 μm. [Color figure can be viewed in the online issue, which is available at wileyonlinelibrary.com.]

Next, we examined whether HuCNS‐SC differentiated into neurons or glia by labeling with the human specific antibody STEM121 in conjunction with the immature neuronal marker doublecortin and the astrocytic marker GFAP or the human nuclear marker, Ku80 and oligodendroglial markers Olig 2 (Figs. 3 and 4), and GalC (data not shown). In both transgenic models, we observed numerous examples of human cells that were double labeled with STEM121 and doublecortin, particularly when in close proximity to other human cells, whereas only a few GFAP immunopositive astrocytes colocalized with STEM121, regardless of the migration location and distance from the injection core. Colocalization between Ku80 and the oligodendroglial marker Olig2 was also detected (Figs. 3 and 4G–I), whereas expression of GalC, a more mature oligodendrocyte marker, was not detected 6 weeks after transplantation (data not shown).

### Aβ and Tau Pathology is Unchanged by HuCNS‐SC Transplantation in 3xTg‐AD Mice

**Figure 5:**
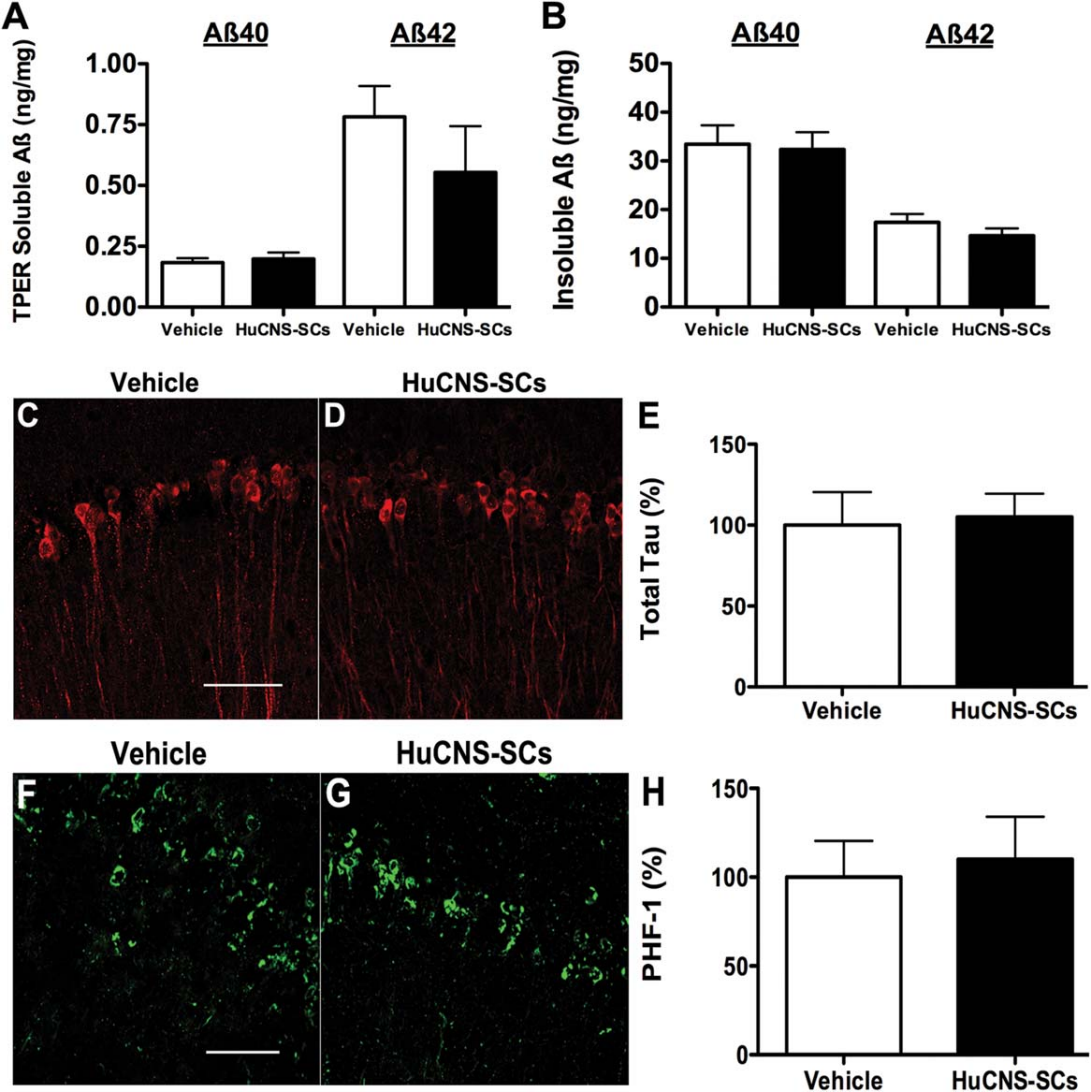
HuCNS‐SC transplantation has no effect on Aβ or tau pathology. ELISA analysis reveals no significant difference in either soluble (A) or insoluble (B) Aβ following HuCNS‐SC transplantation. (C–E) Confocal analysis of total human tau also reveals no difference between vehicle‐ and HuCNS‐SC‐transplanted 3xTg‐AD mice. (F–H) Likewise, levels of hyperphosphorylated PHF‐1 positive tau are unaltered by HuCNS‐SC transplantation. (*N* = 5, data presented as mean ± SEM). Scale bars = 50 μm. [Color figure can be viewed in the online issue, which is available at wileyonlinelibrary.com.]

As changes in Aβ often accompany cognitive changes in AD transgenic mice, we next examined the levels of both detergent‐soluble and insoluble Aβ_40_ and Aβ_42_ using a sandwich ELISA. Interestingly, detergent‐soluble levels of both Aβ_40_ and Aβ_42_ were unaltered between 3xTg‐AD‐vehicle and 3xTg‐AD‐HuCNS‐SC treated mice (Aβ_40_, 0.18 ± 0.02 vs. 0.19 ± 0.03 ng/mg, and Aβ_42_, 0.78 ± 0.13 vs. 0.55 ± 0.19 ng/mg) (Fig. 5A). Similarly, the levels of detergent‐insoluble Aβ_40_ and Aβ_42_ were also equivalent between the two groups (Aβ_40_, 33.75 ± 3.58 vs. 32.65 ± 3.54 ng/mg, and Aβ_42_, 17.41 ± 1.65 vs. 14.65 ± 1.48 ng/mg) (Fig. 5B). These findings are consistent with our previous examination of murine NSC transplantation and suggest that HuCNS‐SC‐induced cognitive improvements occur either independently or downstream of Aβ pathology (Blurton‐Jones et al., 2009).

To evaluate potential changes in tau pathology, we also examined the levels of both soluble and insoluble tau. No changes were found in the steady‐state levels of total soluble tau in 3xTg‐AD‐HuCNS‐SC mice vs. 3xTg‐AD‐vehicle mice (Figs. 5C–E). In addition, immunohistochemical analysis of hyperphosphorylated tau (PHF‐1) showed no differences in CA1 neuronal tau pathology between 3xTg‐AD‐vehicle and 3xTg‐AD‐HuCNS‐SC transplanted groups (Figs. 5F–H).

### Transplantation of HuCNS‐SC Elevate Synaptic Markers in Transgenic Models of Neurodegeneration

**Figure 6:**
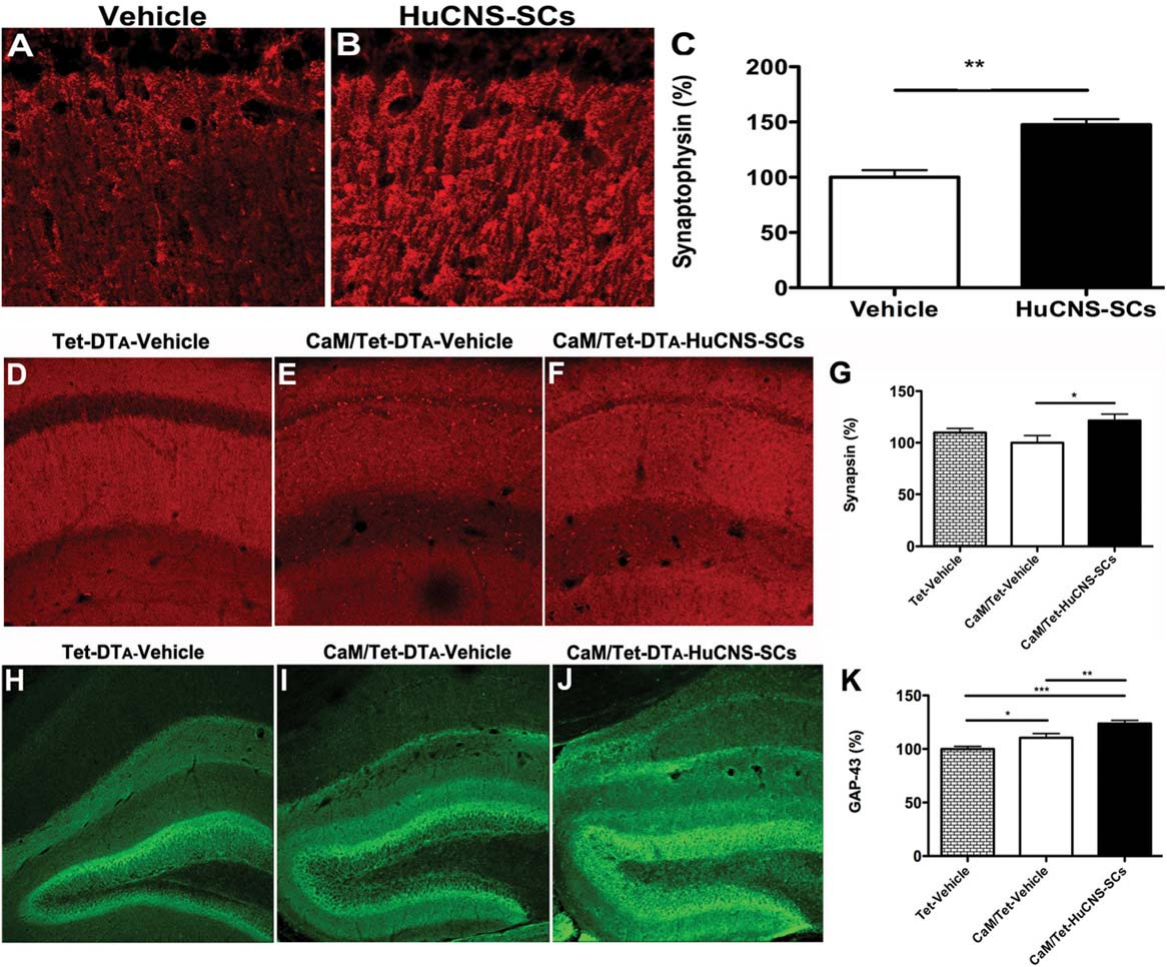
HuCNS‐SC transplantation increases presynaptic markers and axonal sprouting. (A) Immunolabeling of synaptophysin in vehicle injected 3xTg‐AD mice. (B) HuCNS‐SC injection results in a 47% increase in synaptophysin levels in the stratum radiatum of CA1, quantified in (C, *N* = 5, *t*‐test *P* < 0.008). (D) Synapsin in control unlesioned mice reveals a typical pattern of presynaptic innervation within the stratum radiatum of CA1. (E) Surprisingly, synapsin levels are only slightly diminished in lesioned mice. (F) In contrast, HuCNS‐SC transplantation significantly increases the density of presynaptic innervation, quantified in (G, *N* = 8–12, ANOVA *P* < 0.05, Fishers PLSD *P* = 0.016). (H) Compared to control unlesioned mice, we also detected a significant increase in GAP‐43 immunoreactivity within the dentate gyrus of lesioned CaM/Tet‐DT_A_ mice with vehicle injections (I). More importantly, we observed a further enhancement of GAP‐43 expression in lesioned mice transplanted with HuCNS‐SC (J), quantified in (K). (*N* = 10, ANOVA *P* < 0.05, Fishers PLSD *P* = 0.028, *P* = 0.006, *P* = 0.0001). Data presented as mean ± SEM. [Color figure can be viewed in the online issue, which is available at wileyonlinelibrary.com.]

We previously found that increased hippocampal synaptic density is associated with improved cognition after transplantation of murine NSCs (Yamasaki et al., 2007; Blurton‐Jones et al., 2009). To determine whether transplantation of HuCNS‐SC had a similar effect on synaptic connectivity, we examined levels of synaptophysin, synapsin, and growth‐associated protein‐43 (GAP‐43) in 3xTg‐AD and CaM/Tet‐DT_A_ mice. The presynaptic vesicle‐associated proteins synaptophysin and synapsin are frequently used as an indicator of synaptic integrity and are significantly reduced in patients with AD (Sze et al., 1997; Qin et al., 2004). We therefore examined these proteins in HuCNS‐SC transplanted 3xTg‐AD and CaM/Tet‐DTA mice. As shown, we found that the synaptophysin level was significantly increased by 47% in the stratum radiatum of CA1 in HuCNS‐SC transplanted 3xTg‐AD mice vs. Vehicle‐injected mice (Fig. 6C). Similarly, a 21% increase in presynaptic synapsin levels was observed in CaM/Tet‐DT_A_ mice transplanted with HuCNS‐SC (ANOVA *P* < 0.05, Fisher's PLSD *P* = 0.016) (Figs. 6D–G).

GAP‐43 is a neuronal‐specific protein that is localized to axons and involved in synaptic maintenance and neurite growth (Benowitz et al., 1981; Basi et al., 1987; De la Monte et al., 1989). GAP‐43 has also been found to be elevated after CNS injury as a compensational response (Benowitz et al., 1981; Kalil and Skene, 1986). In agreement with this, a significant increase in GAP‐43 was detected after induction of DT_A_ (ANOVA *P* < 0.05, Fisher's PLSD *P* = 0.028; Figs. 6H,I,K). Interestingly, levels of GAP‐43 were further elevated in induced CaM/Tet‐DT_A_ that received HuCNS‐SC, suggesting that NSC transplantation can enhance the axonal sprouting response to injury (ANOVA *P* < 0.05, Fisher's PLSD *P* = 0.006) (Figs. 6I,J,K). No differences in GAP‐43 immunoreactivity were detected in HuCNS‐SC transplanted 3xTg‐AD mice (data not shown).

## DISCUSSION

Alzheimer's disease is one of several neurodegenerative conditions characterized by the presence of insoluble protein aggregates. In AD, aggregation of the Aβ peptide in particular seems to initiate a cascade of detrimental events including the hyperphosphorylation and accumulation of tau, inflammatory activation, and synaptic and neuronal dysfunction and loss. Over the last decade, several classes of drugs aimed at altering Aβ production, aggregation or degradation/clearance have been clinically tested. However, none of these compounds has provided meaningful improvements in cognitive outcome despite their ability to lower Aβ (Hardy and Selkoe, 2002; Karran et al., 2011). As a result, novel approaches should be urgently explored to broaden hypothesis testing by targeting other important disease mechanisms.

The usage of stem cell transplantation as a potential therapy for CNS injury and disease has received increasing attention due to several intrinsic advantages that stem cells may provide over more traditional pharmacological approaches. For example, stem cells from a variety of tissue sources have been shown to produce neurotrophic and other neuroprotective factors, providing a neuroprotective effect that promotes the function and/or regeneration of endogenous neuronal circuitry (Lu et al., 2003; Bakondi et al., 2009). Several of the neurotrophins released by stem cells, including brain‐derived neurotrophic factor, are decreased in the AD brain (Connor et al., 1997; Narisawa‐Saito et al., 1996; Cattaneo et al., 2008). Delivery of neurotrophins and other therapeutic proteins via stem cell transplantation represents an alternative approach to viral gene therapy, which is limited by the small radius of infectivity (<0.5 mm), or direct delivery, which is often associated with harmful side effects such as hyperalgesia (Lewin and Mendell, 1993). In addition to secreting beneficial factors, stem cells can migrate beyond the transplantation site, home to areas of injury (Aboody et al., 2000; Park et al., 2012), and differentiate into damaged or lost CNS cells (Uchida et al., 2012). Thus, NSCs could potentially provide benefits via multiple mechanisms.

In our current experiments, we used human neural stem cells (HuCNS‐SC), derived from donated fetal brain tissue, to test the hypothesis that human NSC transplantation can improve the cognitive decline in AD models that exhibit either Aβ and tau pathology or neuronal loss. By using two complementary transgenic models, 3xTg‐AD and CaM/Tet‐DT_A_ mice, we could more completely assess the impact of HuCNS‐SC transplantation on the cognitive dysfunction that results from AD‐associated proteinopathy and hippocampal neuronal loss. In this study, we show that HuCNS‐SC transplantation successfully rescues the cognitive decline associated with both of these transgenic models. To determine the behavioral effects of HuCNS‐SC transplantation, 3xTg‐AD or CaM/Tet‐DT_A_ were subjected to three distinct hippocampal dependent behavioral tasks. Transplantation of HuCNS‐SC resulted in significant improvements in hippocampal‐dependent cognitive function in both 3xTg‐AD and CaM/Tet‐DT_A_ mice. The intriguing observation that HuCNS‐SC transplantation improves MWM probe trial and object recognition performance in 3xTg‐AD mice but has no effect on MWM acquisition, suggests that NSCs may specifically enhance processes involved in memory consolidation, but may have less of an effect on learning of the task.

Interestingly, HuCNS‐SC transplantation had no effect on Aβ or tau pathology, suggesting that the mechanism of action occurs downstream from these pathologies. Indeed, HuCNS‐SC transplantation does appear to modulate hippocampal synaptic connectivity in 3xTg‐AD and CAM/Tet‐DT_A_ mice as evidenced by increased levels of synaptic and growth‐associated proteins. Thus, the loss of synaptic connectivity, which correlates more closely with cognitive dysfunction than either Aβ or tau (Terry et al., 1991), is modulated by HuCNS‐SC transplantation. The mechanism of action for this modulation is currently unknown; nevertheless it is reasonable to speculate, given the supportive murine NSC data in the same animal models, that it may involve BDNF, as HuCNS‐SC produce physiologically relevant levels (59.1 pg/mg) of this neurotrophin (McGill et al., 2012). Interestingly, the HuCNS‐SC‐mediated impact on cognition and synaptic and growth associated markers occur within a relatively short time post‐transplantation (4–6 weeks), when most donor cells are still relatively immature, suggesting that a specific differentiation program may not be necessary for efficacy; rather, intrinsic properties of HuCNS‐SC may be more relevant in these models.

Our lab has previously used allogeneic murine NSCs in proof of concept studies of a therapeutic strategy in AD (Blurton‐Jones et al., 2009). In the current study, we sought to determine whether research grade HuCNS‐SC cells were efficacious when transplanted into transgenic models as a first step toward the potential future testing of Good Manufacturing Practices (GMP)‐compliant human NSCs for AD. Although debate continues as to which type of stem cell should be used to clinically treat patients afflicted with CNS injury, NSCs have proven to be effective in treating several preclinical injury models, and have the potential to differentiate into appropriate CNS relevant cell types. Neural progenitors derived from fetal brain tissue have already been transplanted into the brains of Huntington's disease and Parkinson's disease patients and have shown some promise (Freeman et al., 2000; Mendez et al., 2005; Lindvall and Kokaia, 2006).

In transitioning from murine NSC transplantation to the transplantation of human NSCs in the injured/diseased murine CNS, some researchers have had the advantage of using immune‐deficient mice, which circumvent the issues of immune rejection (Anderson et al., 2011). Immune‐deficient AD models are not currently available; consequently, immunosuppression paradigms must be used to allow the survival and engraftment of xenogenic transplants. Despite this, some studies have transplanted human stem cells into brains of immune competent rodents without any means of immunosuppression (Riess et al., 2002). This approach likely results in the rapid rejection of the transplanted cells, greatly complicating the interpretation of preclinical studies. In addition, acute immune activation has been previously shown to reduce pathology in AD murine models (DiCarlo et al., 2001; Herber et al., 2007). Thus, any benefits observed from human NSC transplantation into nonimmunosuppressed mice would be difficult to interpret. To prevent the cell rejection that normally results from xenogeneic transplantation, we systemically administered the conventional suppressant tacrolimus (FK506) to CaM/Tet‐DT_A_ with the addition of an antibody against CD4 to suppress T cell response. FK506 and CD4 treatments were well tolerated by CaM/Tet‐DT_A_ mice over the 6‐week experimental period. The treatment of transgenic AD mice with FK506 has been shown to differentially effect the development of Aβ and tau pathology (Yoshiyama et al., 2007; Luo et al., 2008; Hong et al., 2010). Hence, we instead used a recently described T cell costimulatory suppression regimen for our studies in 3xTg‐AD mice (Pearl et al., 2011).

Full GMP HuCNS‐SC that are similar to the research grade lot used in the current study have already been successfully tested in Phase I studies (Gupta et al., 2012; Selden et al., 2013). These initial trials in patients with Batten's disease and Pelizaeus‐Merzbacher Disease (PMD), have shown that of HuCNS‐SC are well‐tolerated, engraft into the human brain for up to two and a half years (1.5 yrs postimmunosuppression), and demonstrate a favorable safety profile (Gupta et al., 2012; Selden et al., 2013). Despite our promising findings, it will be critical to also examine fully GMP‐compliant NSCs in appropriate animal models before proceeding to potential early stage clinical trials.

Taken together, our current data demonstrate for the first time that the human NSCs can improve cognition in two complementary models of AD pathogenesis and hippocampal neuronal loss. We find that HuCNS‐SC have no effect on Aβ and tau pathology, but rather increase key markers of synaptic plasticity and growth. Thus, hNSC transplantation may offer a novel and promising approach to treat AD that warrants further translational investigation.

## Supporting information

Supplementary Information
