# Supplementary figures and images for: Human neural stem cells improve cognition and promote synaptic growth in two complementary transgenic models of Alzheimer's disease and neuronal loss
Source: Hippocampus. 2015 Jan 8;25(7):813–26. doi: 10.1002/hipo.22405 (PMC4722865; doi:10.1002/hipo.22405)

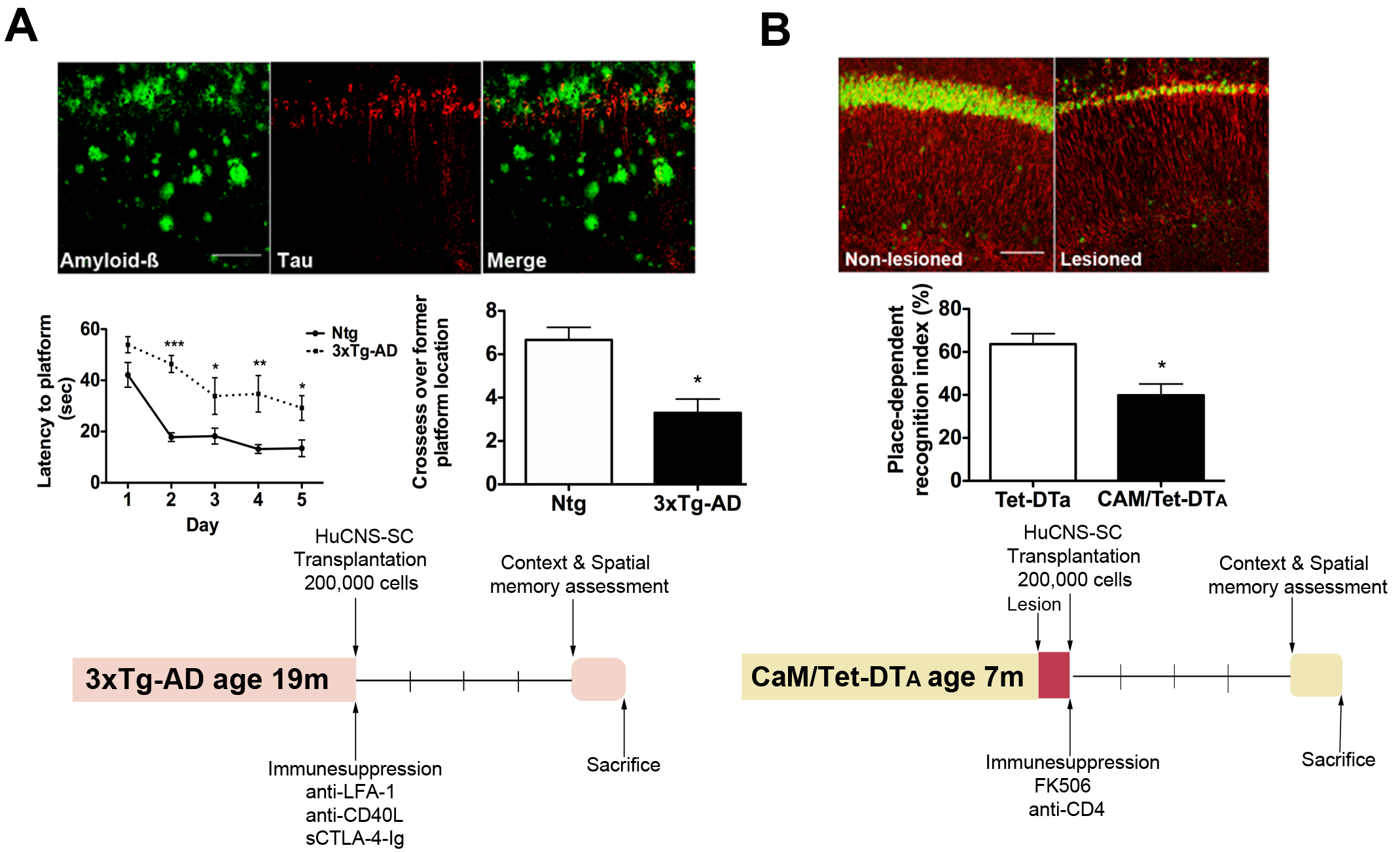

Supplement: Supplementary file 1 — Supplementary Information [file HIPO-25-813-s001.tif]
